# Advances and challenges in the development of visual prostheses

**DOI:** 10.1371/journal.pbio.3002896

**Published:** 2024-10-24

**Authors:** Eduardo Fernandez, Jose Antonio Robles

**Affiliations:** 1 Bioengineering Institute and Cátedra Bidons Egara, University Miguel Hernández, Elche, Spain; 2 CIBER Research Center on Bioengineering, Biomaterials and Nanomedicine (CIBER BBN), Madrid, Spain; 3 John Moran Eye Center, University of Utah, Salt Lake City, Utah, United States of America; 4 Radboud University, Donders Centre for Neuroscience, Nijmegen, the Netherlands

## Abstract

Visual prostheses have undergone significant developments in the past 20 years, from early retinal implants to recent cortical approaches, but their clinical application is still limited. This Perspective outlines the remaining challenges to achieve the ambitious clinical goals for visual prostheses.

## Teaser

The past 20 years have witnessed significant advancements in the field of visual prostheses, with developments spanning from early retinal implants to recent cortical approaches. This Perspective looks at some of the remaining challenges to achieve the ambitious clinical goals that these technologies could enable.

Visual impairment profoundly impacts individuals’ lives, compromising personal autonomy and diminishing overall quality of life. Over the last 2 decades, the field has progressed from experimental concepts to clinically available devices [1]. This evolution has yielded varying degrees of success, reflecting both advances made and the challenges that remain.

The early 2000s marked the beginning of tangible progress in visual prostheses development, particularly with the emergence of retinal implants. The Argus II Retinal Prosthesis System, developed by Second Sight, was one of the first devices to receive regulatory approval [2]. The Alpha IMS developed by Retina Implant AG also made significant strides [3]. These early systems provided users with a limited form of vision, allowing them to perceive light, shapes, and motion. However, despite the initial promising results, the devices ultimately fell short of unmet expectations, leading to their eventual withdrawal from the market [4]. This outcome underscores the complexity of developing visual prostheses and highlights the need for continued research and development in the field.

Many of the challenges in the field of visual prostheses are shared with the broader field of neurotechnology and span various aspects of neural interface development and implementation, reflecting the interconnected nature of neuroscientific research and technological innovation [5]. To be able to stimulate individual neurons, the microelectrodes should be located very close to target cells and have dimensions similar to the neurons they are trying to stimulate. Likewise, both the electrodes and the substrates housing the connecting pathways must be highly biocompatible and completely insulated. This is crucial to mitigate the foreign body response, prevent degradation, and minimize crosstalk between electrodes. Furthermore, it is important to ensure the mechanical stability of implanted devices over time. Additionally, a significant challenge is the limited ability of current devices to constrain electrical fields to target specific cell types or structures. All these requirements impose unique constraints on the architecture and materials used in the design and implementation of visual prostheses.

A more specific challenge of visual prostheses lies in the complexity of the human visual system. The current status of these devices is in stark contrast to that of cochlear implants (hearing restoration prostheses), which provide reliable speech recognition to many deaf patients, even though they reduce approximately 30,000 auditory axons to no more than 12 to 32 stimulating electrodes. This is largely attributed to advances in signal processing and encoding techniques that mimic frequency encoding in the cochlea [6]. However, despite significant progress in the past few years, visual prostheses still lack the complex processing capabilities of the visual system.

The human eye clearly surpasses the complexity of the auditory system, containing approximately 125 million photoreceptors that respond to light and includes complex neural circuits for processing visual information. The output neurons of the eye are the retinal ganglion cells, which send approximately 1 to 1.5 million axons via the optic nerve to the brain [7]. Thus, to faithfully replicate the human retina’s capacity for encoding the multifaceted features of objects in the visual field—such as form, localization, texture, contour, and color—a prosthetic device would require at least 1 million parallel channels. Consequently, replicating even a small fraction of visual system functionality with an artificial device is a difficult task that exceeds the capabilities of current technologies.

Even if only a rudimentary representation of the surrounding physical world can be evoked, a blind individual could use this artificial information to carry out activities of daily living. Several simulation studies indicate that it is feasible to perform basic visual tasks, such as reading and navigating through unfamiliar environments, using only 600 to 700 electrodes [8]. However, this limited number of electrodes usually implies a reduced visual field, which can pose significant challenges for tasks such as orientation and mobility. Consequently, the development of new systems that integrate a greater number of electrodes and are capable of providing larger visual fields with different levels of processing depending on the implantation site would be essential for restoring even basic functional vision. Nevertheless, simply increasing the number of stimulating electrodes may result in perceptually meaningless outcomes. The problem lies in transmitting biologically meaningful information to the appropriate neural sites.

**Fig 1:**
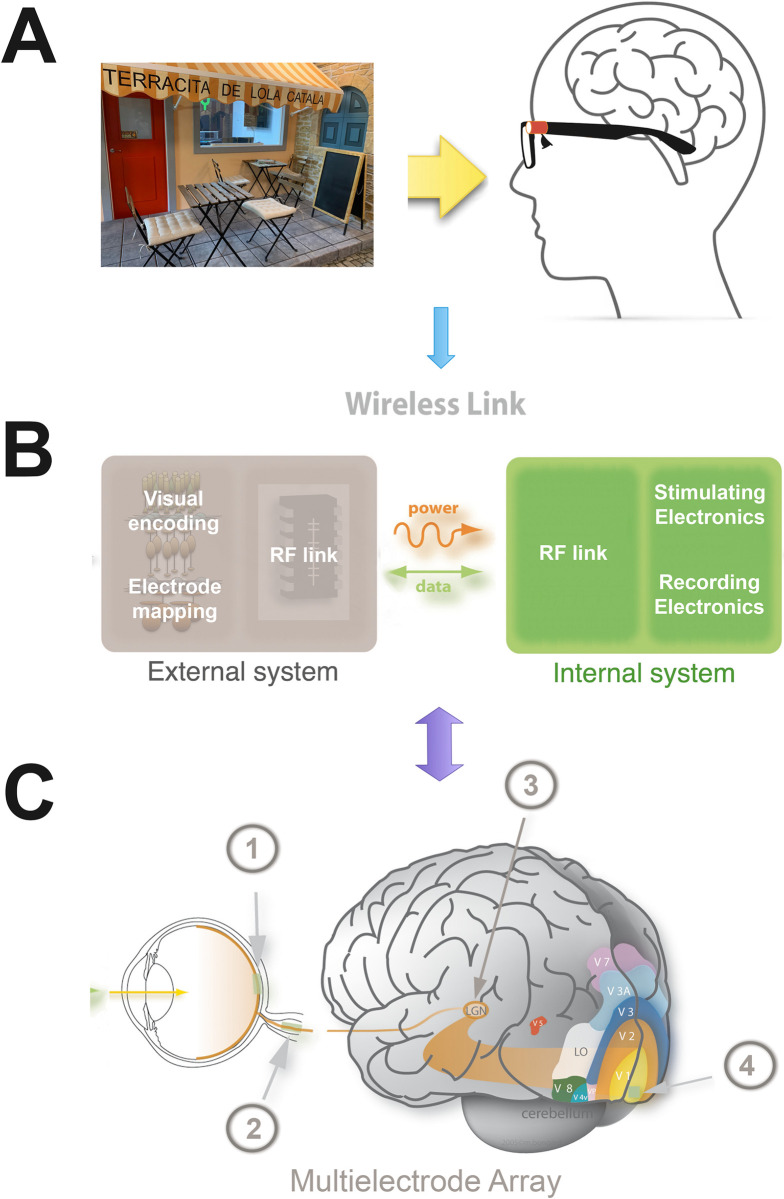
Design of visual prostheses. All approaches generally share a common set of key components. (**A**) A camera, typically mounted on standard glasses, captures the subject’s visual field. (**B)** An external processing unit extracts the most relevant features from the environment, converting visual scenes into patterns of electrical stimulation. This unit wirelessly transmits power and data via a radiofrequency (RF) link to the internal implanted system, which decodes the signals, identifies the target electrodes, and generates the final stimulation waveforms. In some designs, the internal system also supports electrophysiological recordings. (**C)** Finally, a multi-electrode array is implanted at one of several potential sites along the visual pathway, including the retina (1), optic nerve (2), lateral geniculate nucleus (3), or visual cortex (4).

While the retina extracts complex visual features such as shape, color, contrast, and motion, current visual prostheses strategies focus predominantly on addressing only spatial resolution. This suggests that in addition to image resolution, future visual prostheses should also pay attention to other relevant attributes such as receptive field size, motion, temporal encoding, etc. Furthermore, because vision is an active process and static images stabilized on the retina fade quickly, preprocessing should incorporate the jerk-like movement of the eyeballs, which subserves vision by abruptly changing the point of fixation to prevent this fading. Additionally, the location of evoked phosphenes can be shifted based on the eye position at the time of electrical stimulation [9]. This is especially relevant for visual prostheses intended to be located outside of the eye, such as devices designed for placement at the optic nerve, lateral geniculate nucleus (LGN), or primary visual cortex (Fig 1).

There are also significant problems related to power and data transmission. The integration of wireless technologies has transformed the way visual prostheses are powered and controlled, enhancing the safety and reliability of many current devices. Nonetheless, there are still power and communication limitations, along with the challenge of power dissipation, particularly when a large number of electrodes are involved. Consequently, there is a clear need for the development of new technologies optimized for high channel counts. Furthermore, we should consider the development of new tools to enhance bidirectional interaction with the targeted neurons. This approach has been successfully used to optimize stimulation parameters in retinal and cortical prostheses and could be decisive for the development of next generation visual prostheses [10].

Another critical factor for the clinical success of future visual prostheses is the active involvement of clinicians and end users throughout all phases of development and deployment. Their insights are invaluable to improve the design and obtain essential information regarding everyday challenges, preferences, and expectations. At the same time, we should consider the variability in patient outcomes. While some patients experience meaningful improvements in vision, others have little benefit from their implants. Understanding the factors that contribute to these differences is crucial for developing more effective and personalized systems. The success of any visual prostheses largely depends on the user’s ability to adapt to the artificial visual input, which represents a different form of vision compared to natural sight.

The application of advanced artificial intelligence techniques and machine learning algorithms could significantly help users to interpret and use phosphene-based visual information. Additionally, integrating visual prostheses with other sensory modalities, such as auditory or tactile feedback, could provide a more comprehensive sensory experience.

Developing a visual prosthesis is an expensive endeavor, requiring extensive research, clinical trials, and regulatory approval. The high costs associated with these procedures have placed a significant financial burden on companies in the field. Developing cost-effective solutions and ensuring that these technologies are available to a broader range of patients will be a key to their long-term success. In addition, we should establish rigorous standardized criteria for the indications of each device, patient selection, and determining the optimal time for implantation in each subject.

We foresee that collaboration between neuroscientists, engineers, clinicians, and end users is essential to overcoming the multifaceted challenges of visual prostheses development. As the field advances, it will also be important to address the ethical considerations surrounding visual prostheses, particularly in terms of use, accessibility, equity, psychological effects, long-term effects, and safety. We anticipate that advancements in implanted neural technologies, materials, neuroscience, and electronics, along with enhanced intelligence in these systems, will pave the way for a new generation of devices capable of restoring functional vision for many blind patients.
